# Traumatic Orbital Apex Syndrome With Acute Orbital Compartment Syndrome Secondary to Facial Fractures: A Case Report

**DOI:** 10.7759/cureus.100651

**Published:** 2026-01-02

**Authors:** Diogo R Branco, Diogo Conduto, Vasco Lobo, Gaizka Ribeiro, Miguel Andrade

**Affiliations:** 1 Department of Plastic and Reconstructive Surgery, Hospital Santa Maria, Unidade Local de Saúde Santa Maria (ULSSM), Lisbon, PRT; 2 Department of Ophthalmology, Hospital Santa Maria, Unidade Local de Saúde Santa Maria (ULSSM), Lisbon, PRT

**Keywords:** case report, facial trauma, orbital compartment syndrome, surgical decompression, traumatic orbital apex syndrome, zygomaticomaxillary complex fracture

## Abstract

Orbital apex syndrome (OAS) is a rare yet severe complication of facial trauma, characterized by acute visual loss and ophthalmoparesis due to optic neuropathy and involvement of the superior orbital fissure’s (SOF) emerging structures. When associated with acute orbital compartment syndrome (AOCS), OAS constitutes a medical emergency due to the high risk of irreversible visual loss, requiring emergent fracture reduction and decompression.

We present a case of a 39-year-old male admitted following facial and cranioencephalic trauma with loss of consciousness after a fall from a 3 m height. On admission, he exhibited proptosis, eyelid ptosis, anisocoria with fixed mydriasis, ophthalmoparesis, decreased visual acuity, and elevated intraocular pressure. CT imaging revealed a left zygomaticomaxillary complex (ZMC) fracture with orbital floor involvement, SOF narrowing due to fracture fragments, and ocular globe tenting. A diagnosis of OAS and AOCS secondary to SOF narrowing was made, and the patient underwent emergent fracture reduction and fixation with immediate intraoperative decompression. Postoperatively, the patient demonstrated favorable evolution with resolution of proptosis, full recovery of ocular motility, and full visual recovery. To our knowledge, traumatic orbital apex syndrome associated with acute orbital compartment syndrome has been rarely reported in the literature, and this case illustrates the potential benefits of prompt, coordinated multidisciplinary intervention. Nevertheless, conclusions drawn from a single case must be interpreted with caution, as the rarity of this condition limits the availability of high-level evidence and precludes definitive treatment guidelines.

## Introduction

The orbital apex is the posterior region of the orbit, anatomically defined as the convergence of the optic canal and the superior orbital fissure (SOF). This area contains critical neurovascular structures, including the optic nerve (CN II), oculomotor nerve (CN III), trochlear nerve (CN IV), abducens nerve (CN VI), and the ophthalmic division of the trigeminal nerve (CN V1 - frontal, lacrimal, and nasociliary nerves), along with the ophthalmic artery and superior ophthalmic vein and central retinal pedicle [1,2]. The confined space of the orbital apex, combined with the presence of critical neurovascular structures, makes even minimal structural displacement or increases in intraorbital pressure clinically significant, often requiring urgent intervention to prevent permanent visual loss due to neural and vascular compromise [1-4].

Orbital apex syndrome (OAS) refers to a constellation of signs and symptoms resulting from pathology in this region, as follows: (1) acute vision loss due to optic neuropathy, (2) ophthalmoparesis, (3) eyelid ptosis, and sometimes (4) sensory deficits in the V1 distribution [1,3]. Clinically, OAS overlaps with superior orbital fissure syndrome (SOFS) and cavernous sinus syndrome, but the involvement of the optic nerve is a defining feature of true OAS [1,5].

The etiology of OAS is heterogeneous and includes inflammatory (e.g., Tolosa-Hunt syndrome, vasculitis), infectious (bacterial, fungal), neoplastic (head and neck cancers with perineural spread, lymphoma), vascular (carotid cavernous fistula, cavernous sinus thrombosis), and endocrine (thyroid orbitopathy) causes [1,2]. Traumatic OAS is a rare but significant etiology, typically resulting from craniomaxillofacial trauma causing direct compression of the optic nerve and adjacent cranial nerves by displaced bone fragments, hematomas, or orbital edema. Previous studies have demonstrated that even relatively small midfacial or orbital fractures may result in optic nerve compromise when the orbital apex is involved [6,7].

When OAS is associated with acute orbital compartment syndrome (AOCS), prognosis deteriorates significantly. Elevated intraorbital pressure reduces perfusion of the optic nerve and central retinal circulation, leading to optic nerve ischemia and rapid, potentially irreversible visual loss if decompression is delayed [1,8,9]. Experimental and clinical evidence suggests that sustained elevation of intraorbital pressure compromises axoplasmic flow and vascular supply to the optic nerve, explaining the narrow therapeutic window observed in these patients [1,9]. This pathophysiological mechanism highlights why AOCS represents a true ophthalmological emergency.

Despite its clinical severity, traumatic OAS remains poorly represented in the literature, with most evidence limited to isolated case reports and small case series [2,3,5]. As a result, there is no consensus regarding optimal management, particularly with respect to the timing of surgical decompression and the choice of surgical approach. Although early decompression is generally advocated when direct nerve compression is radiologically evident, the absence of standardized guidelines continues to generate uncertainty in clinical decision-making [2,3,10].

The presence of concomitant AOCS further complicates management, as traditional anterior decompressive measures, such as canthotomy and cantholysis, may be insufficient when the primary site of compression is posterior, at the level of the orbital apex [8,9]. In such cases, definitive management requires prompt fracture reduction and decompression to relieve posterior orbital pressure and restore optic nerve perfusion [3,5,9]. In this article, we describe a rare presentation of traumatic orbital apex syndrome associated with acute orbital compartment syndrome secondary to zygomaticomaxillary complex and orbital floor fractures, contributing to the limited literature on this vision-threatening condition.

## Case presentation

A 39-year-old male construction worker fell approximately 3 m from a roof, with direct impact of his face and head against the ground. He was found unconscious, later recovered, but remained confused and agitated. On examination, the patient had a Glasgow Coma Scale (GCS) of 13, left-sided facial edema, and periorbital ecchymosis [11]. On ophthalmologic examination of the left eye, visual acuity was limited to light perception. The pupil was fixed and dilated, with a relative afferent pupillary defect. Intraocular pressure (IOP) was markedly elevated in the left eye (32 mmHg) compared with the right eye (12 mmHg). There was proptosis with periorbital edema, ptosis of the upper eyelid, and subconjunctival hemorrhage. Ocular motility was restricted, with limitation of abduction (-3), adduction (-1), supraduction (-3), and infraduction (-2). Slit lamp examination demonstrated preserved integrity of the globes, clear corneas, deep anterior chambers without cells or flare, and a transparent lens. Fundus observation of both eyes was normal.

**Figure 1:**
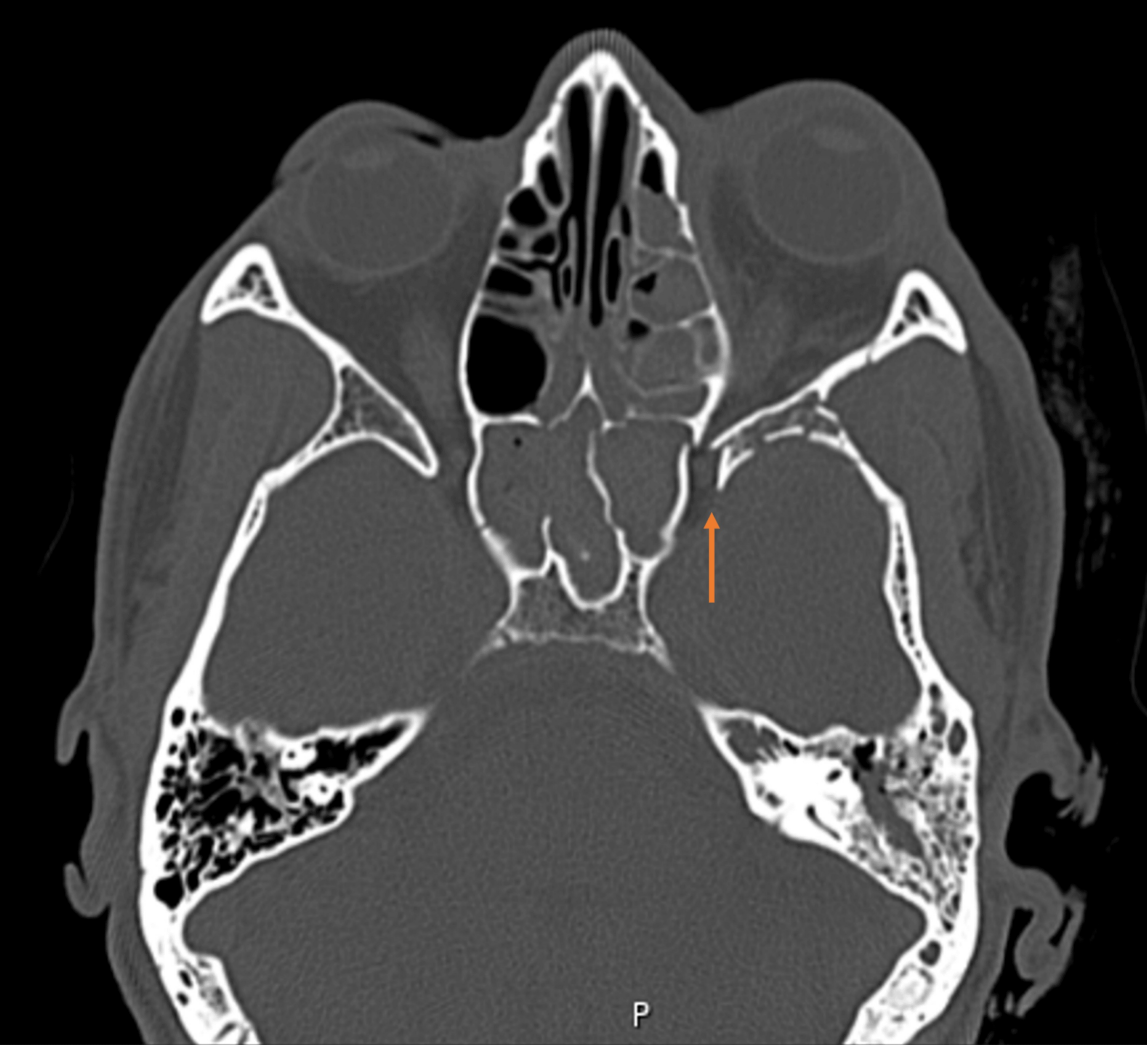
Preoperative CT imaging (axial view). The orange arrow highlights the preoperative narrowing of the superior orbital fissure caused by displaced bony fragments from the zygomaticomaxillary complex (ZMC) fracture.

**Figure 2:**
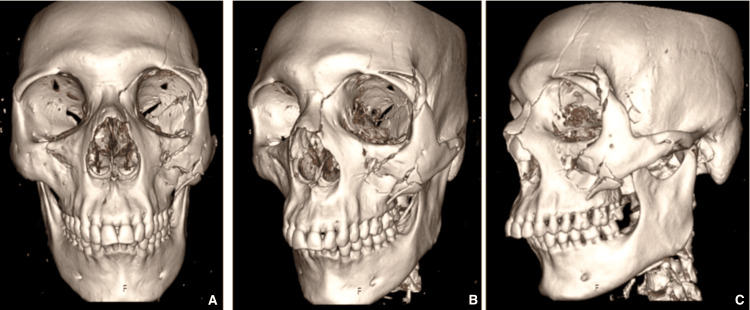
Preoperative CT imaging - 3D reconstruction. The first image (A) corresponds to the anterior-posterior view; the second image (B) corresponds to the left oblique view; and the third image (C) corresponds to the left lateral view.

On imaging, cranioencephalic and maxillofacial computed tomography revealed a left zygomaticomaxillary complex (ZMC) fracture, involving the orbital floor as well as the frontomalar and frontomaxillary pillars (Figures 1, 2A-2C). No incarceration of the inferior rectus muscle or herniation of intraorbital content was noted. A comminuted fracture of the left sphenoid wing and corresponding sinus was also identified, with displaced bone fragments causing significant narrowing of the superior orbital fissure (Figures 1, 2A-2C). There was evidence of left ocular globe tenting and proptosis, without associated retrobulbar hematoma. Intracranially, small fronto-parieto-occipital subdural hematomas were noted bilaterally, with a maximum axial thickness of 4 mm, but without mass effect on the adjacent brain parenchyma. Furthermore, a dissection of the left vertebral artery was observed, extending from the C3 level to the carotid termination. Intracranial circulation appeared to be maintained through probable collateralization. Urgent neurosurgery and vascular surgery consultation recommended hospital admission and vigilance, as well as coordination between both specialties regarding the beginning of anticoagulant therapy.

Given the combination of proptosis, fixed mydriasis, ophthalmoparesis, reduced visual acuity, relative afferent pupillary defect, radiological evidence of SOF narrowing, globe tenting, and an IOP of 32 mmHg, a diagnosis of traumatic OAS with orbital compartment syndrome secondary to direct compression by displaced fracture fragments was established. For clinical applicability, key red flag features suggestive of traumatic orbital apex syndrome are summarized in Table 1.

**Table 1:** Red flag features for suspected traumatic orbital apex syndrome. The presence of these features following facial trauma should raise suspicion for traumatic orbital apex syndrome and prompt urgent multidisciplinary evaluation. II: second cranial nerve (optic nerve); III: third cranial nerve (oculomotor nerve); IV: fourth cranial nerve (trochlear nerve); VI: sixth cranial nerve (abducens nerve)

Categories	Red flag features
Visual findings	Acute visual loss, relative afferent pupillary defect, fixed or dilated pupil - involvement of cranial nerves II
Ocular motility	Ophthalmoparesis involving cranial nerves III, IV, and VI
Eyelid and globe position	Ptosis, proptosis, globe tenting
Intraocular pressure	Elevated intraocular pressure
Imaging	Superior orbital fissure or orbital apex narrowing, sphenoid wing fractures, and posterior bony impingement
Clinical context	Severe facial or craniomaxillofacial trauma

**Figure 3:**
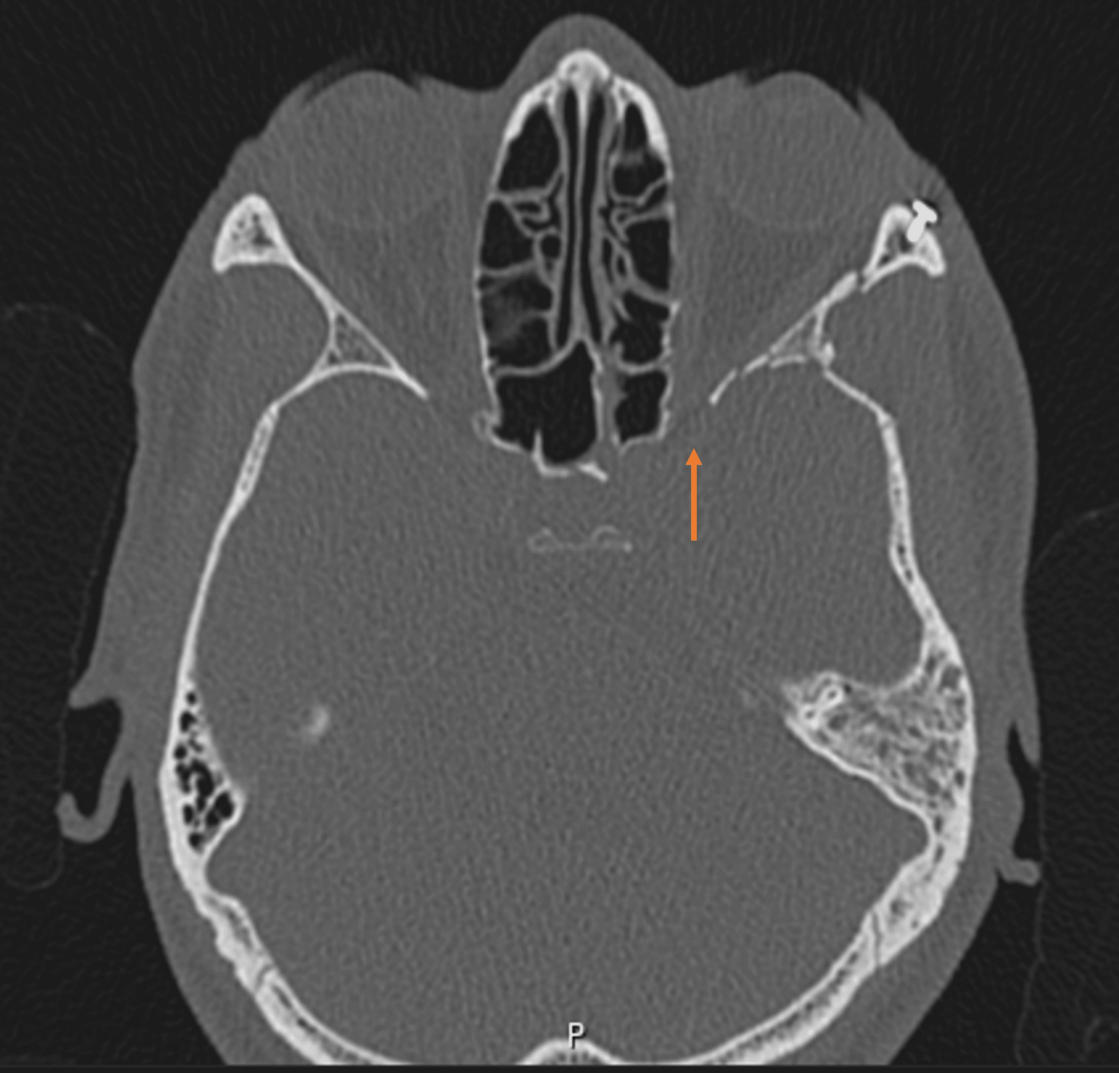
Postoperative CT imaging (axial view). The orange arrow highlights the postoperative normalization of the superior orbital fissure's traumatic narrowing.

**Figure 4:**
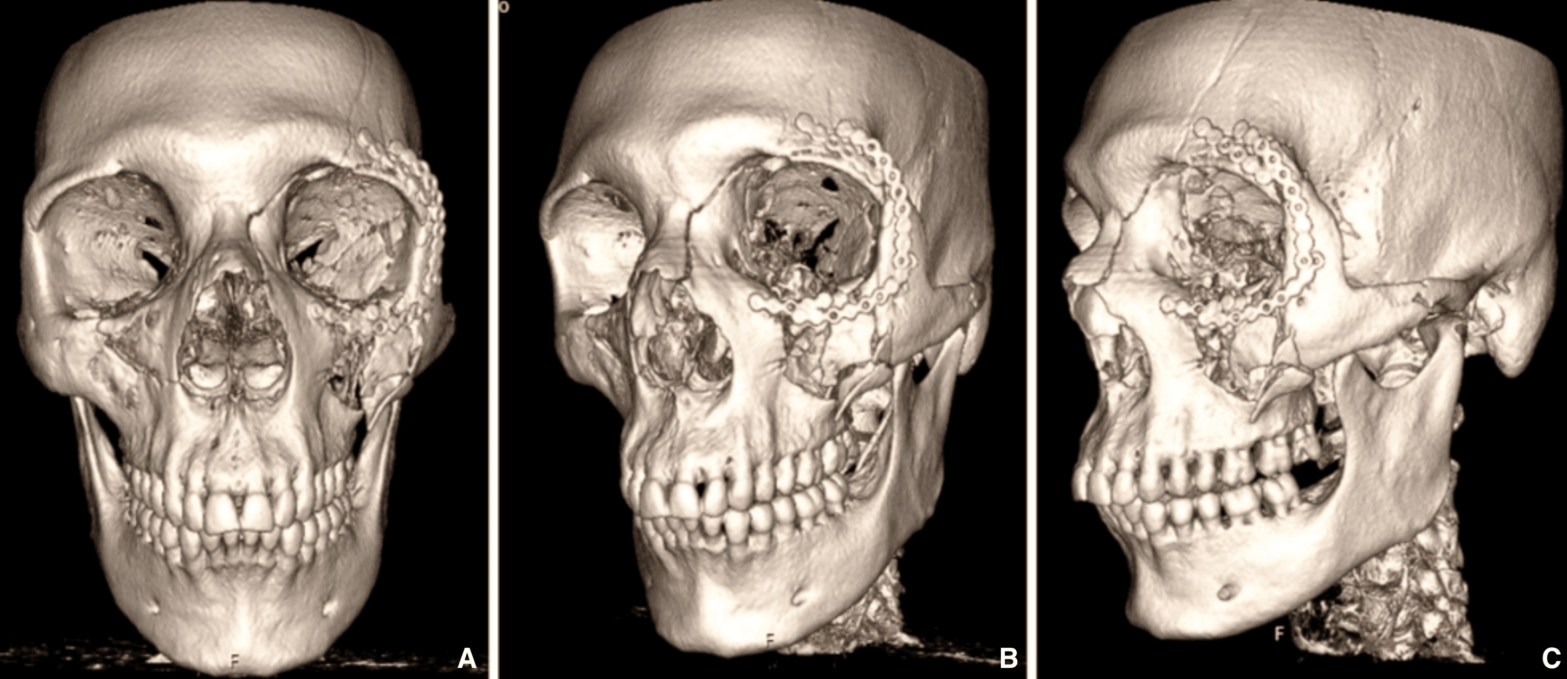
Postoperative CT imaging - 3D reconstruction. The first image (A) corresponds to the anterior-posterior view; the second image (B) corresponds to the left oblique view; and the third image (C) corresponds to the left lateral view.

The patient underwent urgent surgical intervention about 5 h after the accident. A supraciliary (through a traumatic wound extension), subciliary, and upper vestibular approaches were chosen. The zygomatic bone was pushed outward using the joystick technique with an intermaxillary fixation (IMF) screw, and reduction was achieved and maintained through fixation of the frontomalar and frontomaxillary pillars with titanium plates and screws (Figures 3, 4A-4C). Intraoperative ocular pressure measurements normalized (13 mmHg), and intraoperative assessment revealed improvement in proptosis and no restrictions in ocular mobility in the forced duction test.

Perioperatively, the patient received intravenous broad-spectrum antibiotic prophylaxis in accordance with institutional trauma management protocols. The patient’s postoperative course was uneventful, with resolution of proptosis, restoration of full extraocular motility, and partial recovery of visual acuity at discharge (limited to counting fingers at 1 m). The day following surgery, the patient had a control computed tomography scan done, revealing an increased size of the left superior orbital fissure, well-placed osteosynthetic material, absence of ocular globe tenting, but still some persistence of proptosis. Reduction of the thickness of the subdural hematomas was also noted. The findings suggest vertebral dissection remained stable.

Six months after the accident, ophthalmologic evaluation revealed complete visual recovery, with visual acuity of 20/20 in both eyes. The pupils were equal, round, and reactive to light, with no relative afferent pupillary defect. Intraocular pressures were 16 mmHg in both eyes. There was no evidence of proptosis, periorbital edema, or subconjunctival hemorrhage. Eyelid position was normal, with full opening of the left upper eyelid. Extraocular movements were full in all directions of gaze, without restriction. Slit lamp examination revealed a quiet anterior segment with a clear cornea, deep anterior chamber, and transparent lens. Fundus observation of both eyes was normal. A summary of the key events of this clinical case is presented in Table 2.

**Table 2:** Summary of key clinical events and outcomes. CT: computed tomography; GCS: Glasgow Coma Scale; IOP: intraocular pressure; VA: visual acuity; SOF: superior orbital fissure; ZMC: zygomaticomaxillary complex

Time points	Clinical events
Admission (0 h)	Facial and cranioencephalic trauma after 3 m fall; GCS 13; proptosis, fixed mydriasis; VA: light perception; IOP 32 mmHg
Initial imaging	CT scan: left ZMC fracture with orbital floor involvement; sphenoid wing fracture with SOF narrowing; globe tenting
Diagnosis	Traumatic orbital apex syndrome with acute orbital compartment syndrome
Surgery (~5 h post-trauma)	Emergent ZMC fracture reduction and fixation with intraoperative decompression
Day 1 postoperative	CT: increased SOF diameter; well-positioned osteosynthesis material; resolution of globe tenting; normalized IOP
Discharge	Resolution of proptosis and ophthalmoparesis; visual acuity improved to counting fingers at 1 m
6-month postoperative	Complete neurological and ophthalmological recovery; visual acuity 20/20 bilaterally

## Discussion

The diagnosis of OAS is based on clinical features and complemented by additional diagnostic methods, such as laboratory tests and imaging [1]. Involvement of the optic nerve together with any combination of the oculomotor, trochlear, abducens, or ophthalmic branch of the trigeminal nerve (with or without the maxillary branch) is indicative of a true orbital apex syndrome [1,3]. However, it is important to differentiate the diagnosis from the following two different conditions, with overlapping features due to their anatomical proximity: (1) a similar clinical presentation without optic nerve involvement suggests a lesion located at the level of the superior orbital fissure (superior orbital fissure syndrome), (2) if all the features seen in superior orbital fissure syndrome are present, along with involvement of the maxillary division of the trigeminal nerve, one should consider the cavernous sinus syndrome (Table 3).

**Table 3:** Differential diagnosis of orbital apex syndrome (OAS). II: second cranial nerve (optic nerve); III: third cranial nerve (oculomotor nerve); IV: fourth cranial nerve (trochlear nerve); V2: ophthalmic branch of the fifth cranial nerve (trigeminal nerve); VI: sixth cranial nerve (abducens nerve)

Diagnosis	Cranial nerves involved	Cranial nerves spared
Orbital apex syndrome	II+III, IV, VI±V2	-
Superior orbital fissure syndrome	III, IV, VI±V2	II
Cavernous sinus syndrome	III, IV, VI	II, V2

A large variety of pathological processes can cause OAS, such as inflammatory and infectious processes, tumors, endocrinologic conditions, or direct facial trauma. The key aspect to remember while managing a patient with orbital apex syndrome is that treatment is directed towards the underlying condition.

Traumatic OAS is a rare but critical condition requiring prompt recognition and intervention to prevent irreversible visual impairment. In fact, there are only a few cases of OAS reported in the literature, and none had traumatic OAS combined with orbital compartmental syndrome. Current evidence estimates the incidence of superior orbital fissure syndrome to be between 0.3% and 0.8% [6,10], while optic nerve involvement occurs in approximately 0.8-6.0% of cases [7,12]. If one also considers the presence of acute orbital compartment syndrome (AOCS), the incidence is thought to be even lower. In fact, this is the first case report described in the literature of a patient with traumatic OAS with orbital compartment syndrome due to direct bony impingement of the orbital apex structures.

The anatomical confines of the orbital apex, which contain the optic nerve, multiple cranial nerves, and vascular structures, make it susceptible to damage from direct compression by displaced fractures, hematoma formation, and edema in the acute post-traumatic phase [1,3,5]. Traumatic OAS may occur following blunt or penetrating trauma, causing fractures of the orbital walls, leading to direct impingement on the optic nerve and cranial nerves crossing the SOF. Associated orbital compartment syndrome from soft tissue swelling or hemorrhage further exacerbates nerve compression by increasing intraorbital pressure [1-3].

Gossman et al. classified traumatic neuropathy into direct and indirect types [13]. Direct traumatic optic neuropathy refers to cases where there is radiological evidence of compression along the optic nerve pathway, but without complete nerve transection. In contrast, the indirect type is defined by the absence of fractures or any radiographic abnormalities adjacent to the orbital, intracanalicular, or intracranial segments of the optic nerve. This classification has also been applied to superior orbital fissure syndrome [10]. Applying the same distinction to traumatic orbital apex syndrome may also be clinically useful, as the management differs substantially between direct and indirect forms, particularly regarding the urgency and indications for surgical intervention.

This case report highlights the hallmark features of orbital apex syndrome (OAS), including visual loss due to optic neuropathy; fixed mydriasis; ophthalmoparesis with limited extraocular movements caused by dysfunction of oculomotor (CN III), trochlear (CN IV), and abducens (CN VI); proptosis; and upper eyelid ptosis. The case presented is consistent with direct-type traumatic OAS, attributed to direct bony compression of the neurovascular structures at the orbital apex.

However, additional mechanisms likely contributed to the optic neuropathy and subsequent visual loss. These include neurapraxia from traction injury to the optic and oculomotor nerves, and intraorbital inflammation, suggested by elevated intraocular pressure (IOP) and proptosis. Ophthalmoparesis was the result of impaired function of the oculomotor, trochlear, and abducens nerves, consistent with this combined pathophysiology [14]. Ptosis likely resulted from dysfunction of the levator palpebrae superioris muscle (innervated by the oculomotor nerve) and possibly Müller’s muscle, which receives sympathetic innervation [15]. The loss of parasympathetic input to the iris sphincter muscle also resulted in fixed mydriasis [16].

Despite the presence of a blow-out fracture involving the orbital floor, which might otherwise decompress the orbit, direct impingement at the orbital apex has likely compromised venous outflow and contributed to a secondary orbital compartment syndrome. This was exacerbated by intraorbital inflammation and vascular congestion rather than by isolated soft-tissue edema [6,8]. In this context, canthotomy and cantholysis would have been ineffective, as the pressure increase originated posteriorly from direct bone compression rather than from anterior compartment expansion alone [8,9]. Although present, the orbital floor fracture did not mitigate the rise in intraorbital pressure.

The ophthalmic artery, which gives rise to the central retinal artery, courses alongside the optic nerve and supplies the inner retina and the optic nerve head. In some post-traumatic cases, visual loss may result from spasm or occlusion of these arteries. However, in this case, the absence of optic nerve edema and the lack of a "cherry-red spot" on fundoscopic examination argue against vascular compromise as the primary cause of vision loss.

Although the diagnosis of orbital apex syndrome (OAS) is primarily clinical, imaging should be performed in all cases. This is not only due to the high likelihood of associated facial fractures, but also because management strategies differ depending on whether the condition is classified as direct or indirect. Computed tomography (CT) is essential for identifying fractures and assessing narrowing of the superior orbital fissure, whereas magnetic resonance imaging (MRI) may be valuable for evaluating soft-tissue and nerve integrity, particularly in non-traumatic cases of OAS [1,8].

The management of traumatic OAS remains debated, due to the few cases reported in the literature. Current consensus supports prompt surgical decompression in cases of direct-type traumatic orbital apex syndrome, for several key reasons as follows: firstly, the time window for reversing ischemic injury to the optic nerve is narrow, as irreversible damage can occur within just a few hours if intervention is delayed [6,12,17]. Secondly, bony fragments often compromise venous outflow from the orbit and exacerbate compression of the optic nerve and adjacent cranial nerves, contributing to increased tissue edema. Lastly, evidence from case series on traumatic optic neuropathy and superior orbital fissure syndrome indicates that timely surgical management of facial fractures does not compromise visual recovery [6,18].

Surgical intervention, including decompression via fracture reduction and removal of impinging fragments, is the preferred strategy in direct traumatic OAS. Approaches include: (1) transcranial (frontal craniotomy) for deep apex and optic canal decompression in extensive fractures, (2) transorbital approaches (supraciliary, subciliary, transconjunctival) for anterior decompression, (3) combined approaches in complex fractures [1,3,19]. In this case, the reduction of the ZMC fracture resulted in the resolution of the bony impingement of the orbital apex, and restoration of adequate dimension for the superior orbital fissure, consequently normalizing the increased IOP and resolving the acute orbital compartment syndrome. 

The use of high-dose corticosteroids in traumatic OAS remains controversial. Some evidence suggests benefits in reducing edema and secondary ischemic injury [10,12,16], whereas large studies, such as the International Optic Nerve Trauma Study, found no clear advantage over observation or surgery alone [20]. In cases of direct compression, surgery should not be delayed in favour of conservative management. However, high-dose corticosteroids could be started while the patient waits for operating room availability. Although the management of indirect traumatic optic neuropathy lies beyond the scope of this report, high-dose corticosteroids may be considered, even though their therapeutic benefit in this setting remains uncertain.

This report describes a traumatic OAS with orbital compartment syndrome due to direct bony impingement of the orbital apex structures. It supports current evidence demonstrating that prompt surgical decompression can lead to favorable functional outcomes in direct-type traumatic OAS. In the present case, the patient had a worse prognosis since visual acuity was no better than light perception [18]. However, emergent fracture reduction and orbital decompression performed approximately 5 h after trauma resulted in rapid normalization of intraocular pressure, progressive postoperative improvement in visual acuity from light perception to counting fingers at discharge, and complete visual recovery (20/20) at six-month follow-up. These objective outcomes support the role of early surgical decompression in cases of direct bony impingement of the orbital apex.

Nevertheless, conclusions drawn from a single case must be interpreted with caution. The rarity of traumatic orbital apex syndrome, particularly when associated with acute orbital compartment syndrome, limits the availability of high-level evidence and precludes definitive treatment guidelines. Despite these limitations, this case adds to the existing literature by highlighting the potential for full functional recovery when timely, multidisciplinary management is instituted, and underscores the need for further studies to better define optimal treatment strategies and timing of intervention.

## Conclusions

Traumatic orbital apex syndrome associated with acute orbital compartment syndrome secondary to zygomaticomaxillary complex fractures is a rare but vision-threatening condition requiring immediate surgical management. In this case, emergent fracture reduction and orbital decompression performed approximately 5 h after trauma resulted in rapid normalization of intraocular pressure and complete visual recovery (20/20) at six-month follow-up. This case highlights the critical role of a multidisciplinary and rapid-response approach in the management of complex orbital trauma with suspected OAS. Nevertheless, conclusions drawn from a single case must be interpreted with caution, as the rarity of this condition limits the availability of high-level evidence and precludes definitive treatment guidelines.

## References

[1] Badakere A, Patil-Chhablani P (2019). Orbital apex syndrome: a review. Eye Brain.

[2] Gupta R, Khan YA (2015). Traumatic orbital apex syndrome. Can J Ophthalmol.

[3] Yeh S, Foroozan R (2004). Orbital apex syndrome. Curr Opin Ophthalmol.

[4] Imaizumi A, Ishida K, Ishikawa Y, Nakayoshi I (2014). Successful treatment of the traumatic orbital apex syndrome due to direct bone compression. Craniomaxillofac Trauma Reconstr.

[5] Shokri T, Zacharia BE, Lighthall JG (2019). Traumatic orbital apex syndrome: an uncommon sequela of facial trauma. Ear Nose Throat J.

[6] Zachariades N, Vairaktaris E, Papavassiliou D, Triantafyllou D, Mezitis M (1987). Orbital apex syndrome. Int J Oral Maxillofac Surg.

[7] Al-Qurainy IA, Stassen LF, Dutton GN, Moos KF, El-Attar A (1991). The characteristics of midfacial fractures and the association with ocular injury: a prospective study. Br J Oral Maxillofac Surg.

[8] Peter NM, Pearson AR (2010). Orbital apex syndrome from blunt ocular trauma. Orbit.

[9] Gupta D, Beigi B (2017). Orbital compartment syndrome despite significant traumatic expansion of the orbital cavity. Craniomaxillofac Trauma Reconstr.

[10] Chen CT, Wang TY, Tsay PK, Huang F, Lai JP, Chen YR (2010). Traumatic superior orbital fissure syndrome: assessment of cranial nerve recovery in 33 cases. Plast Reconstr Surg.

[11] Teasdale G, Jennett B (1974). Assessment of coma and impaired consciousness. A practical scale. Lancet.

[12] Acartürk S, Seküçoğlu T, Kesiktäs E (2004). Mega dose corticosteroid treatment for traumatic superior orbital fissure and orbital apex syndromes. Ann Plast Surg.

[13] Gossman MD, Roberts DM, Barr CC (1992). Ophthalmic aspects of orbital injury. A comprehensive diagnostic and management approach. Clin Plast Surg.

[14] Pogrel MA (1980). The superior orbital fissure syndrome: report of case. J Oral Surg.

[15] Brent BD, May DR (1990). Orbital apex syndrome after penetrating orbital trauma. Ann Ophthalmol.

[16] Kawasaki AK (2014). Diagnostic approach to pupillary abnormalities. Continuum (Minneap Minn).

[17] Urolagin SB, Kotrashetti SM, Kale TP, Balihallimath LJ (2012). Traumatic optic neuropathy after maxillofacial trauma: a review of 8 cases. J Oral Maxillofac Surg.

[18] Wang BH, Robertson BC, Girotto JA, Liem A, Miller NR, Iliff N, Manson PN (2001). Traumatic optic neuropathy: a review of 61 patients. Plast Reconstr Surg.

[19] Liu J, Zhao J, Wang Y (2021). Simultaneous endoscopic endonasal decompression of the optic canal, superior orbital fissure, and proper orbital apex for traumatic orbital apex syndrome: surgical anatomy and technical note. Front Surg.

[20] Levin LA, Beck RW, Joseph MP, Seiff S, Kraker R (1999). The treatment of traumatic optic neuropathy: the International Optic Nerve Trauma Study. Ophthalmology.

